# Spermatozoa retrieval for cryopreservation after death

**DOI:** 10.1590/S1677-5538.IBJU.2017.0249

**Published:** 2018

**Authors:** Fernando Lorenzini, Eduardo Zanchet, Gustavo M. Paul, Ricardo T. Beck, Mariana S. Lorenzini, Elisângela Böhme

**Affiliations:** 1 Centro de Reprodução Humana Curitiba, PR, Brasil

**Keywords:** Cryopreservation, Spermatozoa, Vas Deferens, Testis

## Abstract

**Objectives:**

To describe the retrieval spermatozoa technique for cryopreservation after death, including the proximal part of vas deferens.

**Material and Methods:**

A 28-years old man, with previous history of infertility, who had died 12 hours before, was submitted to spermatozoa retrieval for cryopreservation, with surgical bilateral resection in bloc of the proximal part of vas deferens, testicle and epididymis. At the laboratory, by milking the epididymis and vas deferens, the extracted fluid was collected; also, three samples of each testicle parenchyma were also harvested.

**Results:**

The fluid from the vas deferens showed spermatozoa, mostly with in situ motility. Testicular fragments also presented spermatozoa, mostly with small tail movements or immobile.

**Conclusion:**

The inclusion of the proximal part of vas deferens during spermatozoa retrieval after death must be performed, since it contains high concentration of spermatozoa, and even in the presence of previous infertility, as was with this patient, it is possible to retrieve spermatozoa.

## INTRODUCTION

Cryopreservation of spermatozoa after death allows parenthood using assisted reproductive technologies.

The success of spermatozoa retrieval after death depends on the time from death to cryopreservation, on the quality of previous spermatogenesis and the harvested anatomic structures. Post-mortem viability is inversely proportional to the time from death to sperm retrieval (1).

The first report on sperm retrieval after death was published in 1980. It describes the sperm retrieval of a 30-years old man with encephalic death due to trauma (2). In 1993, it was reported the birth of the first child using cryopreserved spermatozoa collected after death (3).

The objective of the present paper is to describe the employed technique for posthumous sperm retrieval and cryopreservation including the proximal part of the vas deferens during surgical collection.

## MATERIALS AND METHODS

A 28-years old white man, that had died 12 hours before o was submitted to sperm retrieval for cryopreservation requested by his wife.

Before the procedure, a written consent was signed.

Surgical technique of sperm retrieval used the following sterile surgical steps: patient in dorsal decubitus, antisepsis of genital skin, sterile drapes protecting the surgical field, 4cm longitudinal median incision of scrotal pouch, opening of bilateral vaginal layer, in bloc bilateral resection of proximal 5cm of vas deferens, testicle and epididymis, closure of incision with continuous suture in two layers. Each of the two tissue resected parts was separately involved in sterile drapes, stored in a plastic sterile bag of 15 liters and both were kept in a refrigerated box for biological transport.

For cryopreservation of biological tissue, at the reproduction laboratory, under sterile conditions, it was performed milking of epididymis and vas deferens, and the collected fluid was added to 3mL of culture media in a test tube (single step medium, Irvine Scientific^®^, Santa Ana, EUA). Also, three samples of 3×3×3mm of testicular parenchyma of each testicle (superior, medium and inferior anterior region) were harvested and immersed in a vial with the same culture media. The fluid of the vas deferens ends and the testicle fragments were cryopreserved in liquid nitrogen, using the fast freezing technique (4), with the aid of a cryoprotector media (test-yolk buffer com glicerol, Irvine Scientific^®^, Santa Ana, EUA).

For microscopic analysis, three other samples of the fluid of both vas deferens were collected (20 microliters each), along with three other small resected samples of testicular parenchyma (1×1×1mm each), from the superior, medium and inferior region.

## RESULTS

The proximal part of vas deferens, testicles and epididymis showed macroscopic normal characteristics. Microscopic evaluation of the collected fluid from vas deferens showed the presence of rare spermatozoa in each optic field (200X), mostly with in situ motility. Testicular fragments also presented spermatozoa, mostly with small tail movements or immobile.

## DISCUSSION

Sperm cryopreservation and assisted reproductive technologies allow posthumous parenthood (5). Family members may express this wish and seek help from trained professionals for post mortem cryopreservation of retrieved sperm, as soon as possible, after death. Usually there is no such team on call for this procedures in these rare occasions, but they must follow some aligned steps: a) legal aspects (authorization and legal consent); b) choice of biological material to be harvested with the highest probability to contain viable sperm in adequate quantity; c) packaging, transportation and processing of the material at the reproduction laboratory.

Post-mortem gamete cryopreservation legislation in several countries is conflictive or nonexistent. In many occasions, local laws, institutional guidelines and judicial decisions are used (6). World legislation is not consensual regarding legality of the procedure. In France, Germany, Spain, Canada and Sweden the procedure is forbidden, even with previous consent of the deceased (1). In United Kingdom the deceased donor must have previously consented the use of his genetic material after death (7). In Israel, legislation is more liberal: the widow may request the retrieval and use of genetic material (8). In the United States and Australia there are no specific laws; it is recommended that medical societies determine the guidelines for the procedure. Usually these societies recommends a previous consent of the deceased and a minimum interval of six to 12 months between the retrieval and the use in assisted reproduction. These same societies do not recommend the material collection in situations of familiar dispute (9). A question form of semen donors of semen banks in the US showed that 85.9% of donors would agree to the use of their genetic material if they died and among infertile patients 83.8% agreed (6). In Brazil, the Federal Board of Medicine in 1998 recommended that spouses when alive signed a written consent to inform their wish on the destiny of embryos and/or any cryopreserved reproductive material in case of divorce, severe illness of death. Federal justice board defined that to presume paternity, offspring may be obtained by the widow with express authorization of husband when alive, to use his genetic material (10). In the present case, it was requested authorization and written consent, but, almost one year after cryopreservation, the widow asked for discard of all material, signing a written consent form.

Regarding anatomic sites or tissues that must be harvest for sperm retrieval after death, there is no standardization in literature. Usually, it is recommended to harvest testicles and/or epididymis, including the higher possible amount of biological tissue (11). Techniques that use needle percutaneous collection of testicles, epididymis and vas deferens are not ideal, in view of the small quantity of sperm retrieved, although vas deferens needle puncture may present higher success. Electroejaculation may be an alternative for patients with brain death maintained with mechanic ventilation (12, 13). In the present technique, it was surgically collected the testicles, epididymis, proximal parts of vas deferens, that were harvested in bloc to perform the proximal to distal milking maneuver (from the epididymis to the vas deferens) to collect the highest quantity of fluid. The vas deferens present in normal conditions a high amount of mobile and mature sperm (millions) and may reach 100 times of the seminal sperm concentration (14–16) and therefore it is the ideal place to collect sperm, as already shown in a study of sperm collected in live individuals, before and after cryopreservation (16).

Due to the above considerations and the use of the described technique in this paper, when enough quantity of retrieved sperm is collected from the vas deferens ends, it is possible to dismiss the sperm retrieval of epididymis and/or testicles. In the present patient, three fragments of testicle were also harvested, due to the fact that the patient had a previous history of infertility and the sperm concentration at the fluid from vas deferens was low.

Regarding the time interval between death and sperm retrieval, it should be the shortest possible to obtain live spermatozoa with good motility, and it is suggested that it must not exceed 24 to 36 hours (13, 17). But there is a report of efficient sperm retrieval after 48 horas of death (12). In the present case, the elapsed time was almost 12 hours.

Regarding obstetric complications and/or congenital malformations, there are no reports in literature of increase due to the use of posthumous cryopreserved spermatozoa (18).

The quality of previous spermatogenesis is related to the presence of spermatozoa in the post mortem biological material. A study from Australia reported a failed sperm retrieval less than 24 hours after death of a patient with previous infertility (12). In the present patient, the spouse related that her husband was infertile, but the sperm analysis was performed elsewhere, that could not be accessed. The presence of previous husband infertility may explain the low quantity of retrieved sperm, but did not invalidate the employed described technique.

## CONCLUSIONS

Sperm retrieval post mortem using bilateral in bloc ressection of testicle, epididymis and proximal part of vas deferens was efficient. It is suggested that these structures should be used, once they represent the physiological anatomic sites of spermatogenesis, sperm maturation and presence of mature spermatozoa.
